# Hospital Meetings

**Published:** 1903-06-06

**Authors:** 


### HOSPITAL MEETINGS.

### VICTORIA HOSPITAL FOR CHILDREN.

The annual general meeting of this hospital was held on May 20th, Mr. Martin R. Smith presiding.

The Chairman commented, as he had done in past years, on the insufficient amount of general support which the hospital received, and said that at this juncture an appeal was more than ever necessary, when the enlargement of the hospital would necessitate a corresponding increase in expenses. It was calculated that an additional £3,000 per annum would be required to maintain the hospital in a proper state of efficiency. Few realised, the chairman went on to say, how urgently the new wards were needed; there had been an increase of upwards of 400 cases of out-patients, and they were only able to admit 603, which represented about 3 per cent. of the cases presented there, and if the new wards would accommodate three times the original number, instead of little more than twice, they would still be filled to overflowing. A sum of £12,000 was required before the hospital could be declared free of liability, and to obtain this amount they must, said Mr. Smith, break new ground, and not apply once more to those who had helped them so often. It was impossible to believe that a hospital for children, in one of the wealthiest districts of London, could remain long in debt, into which position they had been brought, not by mismanagement or extravagance, but by imperious necessity. The wards were absolutely insufficient, and the hospital unfit for the purposes for which it was used, and therefore enlargement and reconstruction was forced upon them. Support was also much needed for the convalescent home at Broadstairs, which was doing excellent work under the able superintendence of Mrs. Day. At this home the ground floor ward for helpless children required enlargement, but that would have to stand over for the present. The chairman ended by paying a tribute to the services rendered by the secretary, Mr. H. G. Evered, and by the matron, Miss Watson.

The ordinary business of the meeting was then despatched and the proceedings terminated.

From the report it appeared that the financial position of the hospital was far from satisfactory, it having been necessary to add £300 to the already large debt to the bankers.

The annual deficit in the accounts of the convalescent branch at Broadstairs amounted this year to £759. There was a falling-off noted both in the support to the convalescent home and in the donations to the hospital; but it was satisfactory to observe that the ordinary expenditure had decreased although the work of the hospital had increased. H.R.H. Princess Louise, patron of the hospital, laid the foundation stone of the new wards in July last, and presided at a fête held for the purpose of raising funds for the enlargement. It is hoped that the wards will be completed and opened during 1903. The committee are making a special appeal for a further sum of £9,000 to equip the new wards, and also for £3,000 for the adaptation of the old building in order to provide more accommodation for the staff. A hundred new cots are required, costing £4 4s. each; 25 were promised at the meeting by Mr. Martin R. Smith and several other governors agreed to pay for two or three each.

### CHELSEA HOSPITAL FOR WOMEN.

Lord Glenesk took the chair on the occasion of the annual meeting of Governors, held on May 21st.

The Chairman, in moving the adoption of the report, congratulated the hospital on a most prosperous year, and said that its reputation was greatly enhanced, as was proved by the fact that the three great hospital funds had all given increased support, not on account of the distressful condition of the hospital, but in recognition of the good work they had done. During 1902 they had treated 793 in-patients and 2,778 out-patients, and while 307 major operations had been performed the rate of mortality was only 2 per cent., which was as low as it well could be. In view of this fact, it was a mere form proposing votes of thanks to the medical staff, those figures testified of themselves to the extraordinarily skilful way in which the operations were performed. The Chelsea Hospital for Women, Lord Glenesk went on to say, benefited all classes; the very poor who were treated gratuitously, those who were pretty well off who were invited to contribute something towards their support and even those who were able to pay all their expenses, and thus obtained far better nursing than they would have done in their own homes. There was a slight diminution in the subscriptions during the past year owing to deaths of old subscribers. The number of severe cases tended to increase on account of the high reputation the hospital had acquired in dealing with difficult cases. Their generous benefactor "H.M.E.," by whose munificence they had been enabled to erect an expensive fire-escape staircase, had in the last year sent them no less a sum than £2,000 in consequence of the great satisfaction she felt at the state of the hospital. Lord Glenesk then mentioned that Miss Heather Bigg, their matron, of whose services it was impossible to speak too highly, had vacated her post, and her place had been filled by Miss Edith Edwards, who had received her training in this institution and had already proved herself to be an excellent matron. The Convalescent Home at St. Leonards was well supported, and was a most important adjunct to the hospital. The finances of the hospital were in a thoroughly satisfactory condition.

The adoption of the report was seconded by Mr. Henry E. Wright, treasurer.

A resolution re-electing the members of the council was proposed by Countess Cadogan and seconded by Dr. Hugh Fenton, who said how glad they all felt to see Lady Cadogan back again amongst them once more. Dr. Fenton, after eulogising the work done by the council and the efforts they made to acquaint themselves with the wants and difficulties of the hospital, warned the council that the medical staff would still keep on wanting improvements, etc., in order to enable them to perform their work in the best possible way. There was no better advertisement to the work of the hospital, said the speaker, than for everything to be in tip-top order, and he instanced the case of an American doctor who recently visited the hospital and was much struck by the rapidity of their operations. On the occasion of his visit he witnessed seven operations performed in two hours, and gave it as his opinion that in no other hospital in London or America was such a feat possible. The chief credit for this rapidity was due, said Dr. Fenton, to their well-drilled staff of nurses, and he considered the council had made a very wise choice in selecting Miss Edith Edwards to fill the post of matron.

Mr. T. Dyer Edwardes next proposed a vote of thanks to the honorary medical officers and other officials, which was seconded by Major-General Wemyss.

A vote of thanks to the chairman was then proposed by Mr. J. S. Wood, who stated that 25 years ago he had introduced Lord Glenesk to the hospital, since which time he had never turned his back on them. He himself was the only member of the original council now left, having been interested in the hospital for 28 years, since its foundation.

Colonel the Hon. Sir W. J. Colville having seconded the vote, the proceedings came to a close.

### THE ALEXANDRA HOSPITAL.

The thirty-sixth annual meeting of the Alexandra Hospital for Children with Hip Disease was held on Thursday the 21st inst., Mr. A. Bowlby presiding. The business, which was formal, occupied only a few minutes. The announcement was made by the chairman of the generous gift of Mr. Arthur Wood, and members of his family, of a much-needed Convalescent Home at Clandon, near Guildford, to provide accommodation for 20 children. The hospital thus acquired a country branch within easy reach of London, and when it was remembered that many of the patients were under treatment for as long as two years, the value of the extension could hardly be over-estimated. The cost of furnishing the new home, which will be opened early in July by the Lord Bishop of London, falls upon the authorities of the hospital. Additional subscribers to the general fund are urgently needed, and a special appeal is issued with the report for 1902.

